# Overview of Antiviral Drug Therapy for COVID-19: Where Do We Stand?

**DOI:** 10.3390/biomedicines10112815

**Published:** 2022-11-04

**Authors:** Renata Esposito, Davida Mirra, Liberata Sportiello, Giuseppe Spaziano, Bruno D’Agostino

**Affiliations:** 1 Department of Environmental Biological and Pharmaceutical Sciences and Technologies, University of Campania “Luigi Vanvitelli”, 81100 Caserta, Italy; 2 Campania Regional Centre for Pharmacovigilance and Pharmacoepidemiology, 80138 Naples, Italy; 3 Department of Experimental Medicine—Section of Pharmacology “L. Donatelli”, University of Campania “Luigi Vanvitelli”, 80138 Naples, Italy

**Keywords:** COVID-19, antiviral drugs, natural agents, immune system, approved drugs

## Abstract

The vaccine weapon has resulted in being essential in fighting the COVID-19 outbreak, but it is not fully preventing infection due to an alarming spreading of several identified variants of concern. In fact, the recent emergence of variants has pointed out how the SARS-CoV-2 pandemic still represents a global health threat. Moreover, oral antivirals also develop resistance, supporting the need to find new targets as therapeutic tools. However, cocktail therapy is useful to reduce drug resistance and maximize vaccination efficacy. Natural products and metal-drug-based treatments have also shown interesting antiviral activity, representing a valid contribution to counter COVID-19 outbreak. This report summarizes the available evidence which supports the use of approved drugs and further focuses on significant clinical trials that have investigated the safety and efficacy of repurposing drugs and new molecules in different COVID-19 phenotypes. To date, there are many individuals vulnerable to COVID-19 exhibiting severe symptoms, thus characterizing valid therapeutic strategies for better management of the disease is still a challenge.

## 1. Introduction

A pandemic outbreak caused by COVID-19 disease has infected and killed over six million people so far due to the high infectiousness rate, representing one of the main threats nowadays [1]. The genomic instability of severe acute respiratory syndrome coronavirus 2 (SARS-CoV-2) with constant mutation hindered the strain characterization, promoting the fast spread and consequently a public health crisis [2,3]. Indeed, the virus quickly changes for adapting itself to different environments, developing several mutations associated with multiple variants that compromise the efficacy of the vaccines [4,5]. All of this sheds light on an emerging concern, stressing the urgent need to develop safe and effective therapeutic agents for COVID-19 treatment. In this scenario, researchers and physicians from all over the world have been joining their resources to slow down the virus’s spread, developing new antiviral drugs for SARS-CoV-2 and/or testing the anti-COVID efficacy of old drugs by a drug repurposing approach [6,7,8]. The pharmacological treatment targets virus and host proteins to counter viral entry and replication and immune system factors involved in the inflammatory reaction [9]. Currently, the latest COVID-19 treatment guidelines report monoclonal antibodies as prophylactic and therapeutic methods for preventing virus entry into host cells [10] and antiviral drugs to use in the early stage of the disease to act directly on viral replication [11]. Moreover, the cytokine rise associated with lung and systemic inflammation supports the use of immunomodulants to fight the cytokine storm developing into the severe phenotypes of COVID-19 [12,13,14,15]. Finally, adjuvant therapy has proved useful for reinforcing the host’s immune response against infection [16,17]. Unfortunately, despite the availability of several drug treatments and more effective vaccines, the SARS-CoV2 circulation has not yet been stopped; conversely, it is spreading at an accelerated rate. So, new targets and mechanisms to decline the viral load should be found.

Herein, we attempt to bring to light new insights into basic aspects of current therapy, updates in terms of clinical trials and current approval statuses, and focus our attention on potential drug candidates to slow down the virus’s spread.

## 2. Methods

**Figure 1:**
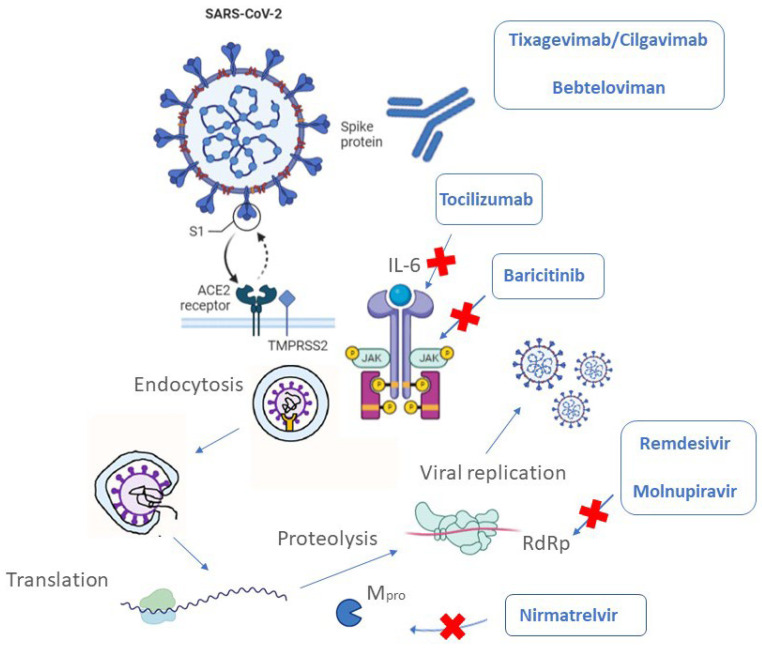
An illustrative picture showing the drugs approved in COVID-19 therapy and their related targets.

PubMed, Scopus, Elsevier and Google Scholar databases were chosen, using the following terms: antiviral drugs, COVID-19 infection, clinical trials and natural products. We performed a deepened research approach for articles from the year 2019 to 2022. Newly published studies were checked weekly until the first week of September 2022. More recently, published studies had a prior evaluation for inclusion. Only articles in the English language were selected. The meta-analysis, clinical trials, cohort studies, brief reports, reviews and systematic reviews were included. Clinical trial data were derived from https://www.clinicaltrials.gov/ (accessed on 13 September 2022). A manual search in the reference list of the included studies was also performed. In general, we followed the PRISMA recommendations for systematic review and meta-analysis documents (Figure 1) [18].

## 3. Therapeutic Agents Approved in COVID-19 Therapy

### 3.1. Monoclonal Antibodies (mAbs)

In the field of a COVID-19 tailored approach, mAbs play an important role in restoring immune homeostasis by a fast and passive immunity response that aids in the destruction of infected cells with the reduction of the viral load [19]. They represent an effective and viable therapy and/or prophylaxis option against COVID-19, associated with a lowering of hospitalizations and death rates, obtaining an emergency authorization for treating mild to moderate COVID-19 subjects [20]. Most of the mAbs targeted the epitopes distributed over S glycoprotein (spike) (S1 and S2) with a high binding affinity by hindering viral entry. In fact, SARS-CoV-2 enters host cells by its spike to bind to the angiotensin-converting enzyme 2 (ACE2) receptor, widely expressed in the respiratory tract [21]. Unlike host proteins, spike is highly mutable and the administration of mAbs against it strictly depends on the circulating variants [22]. In fact, newly emerging SARS-CoV-2 variants harboring mutations can mask the function of neutralizing antibodies, making them less effective [23,24]. The first mAbs approved by FDA are bamlanivimab, casirivimab and imdevimab associated with a lower incidence of hospitalization, emergency department visits and death than the placebo. Overall, their use is well-tolerated and contraindicated in critical patients [25]. Due to emerging variants, the food and drug administration (FDA) has given emergency approval for the mAbs combinations bamlanivimab with etesevimab (BAM/ETE) and casirivimab with imdevimab (REGEN-COV) since the frequency of variants resistant to the mAbs cocktail is lower than a single treatment. Many clinical trials have showed that BAM/ETE and REGEN-COV treatment of COVID-19 outpatients induced improved clinical and virologic outcomes when compared with untreated controls. Notably, the interim analysis of an ongoing clinical trial described the safety and efficacy of REGN-COV in the first 275 non-hospitalized patients with COVID-19. The results revealed the efficacy of the REGN-COV antibody cocktail in enhancing viral clearance and reducing medically attended visits. Interestingly, REGN-COV showed a major efficacy in serum antibody-negative patients or when viral load was higher at baseline [26]. Moreover, a retrospective cohort study determined the real-world effectiveness of REGEN-COV against COVID-19-related hospitalization, severe disease and death [27]. The effectiveness of REGEN-COV was 56.4% in preventing COVID-19 hospitalization, 59.2% in preventing severe COVID-1 and 93.5% in preventing COVID-19 death in the 28 days after treatment. Data suggest that the benefits are greater when REGEN-COV is administered within the first five days following infection. These results were related to Delta variant patients, unlike the Omicron variant which results in less susceptibility. In fact, the Omicron variant (BA.1 lineage) lost susceptibility to the BAM/ETE and REGEN-COV [28]. The spread of the SARS CoV-2 omicron lineages changed the landscape of mAbs activity with respect to previous virus variants. In fact, the emerging BA.2, BA.3, BA.4 and BA.5 lineages showed a 7-16-fold reduction in susceptibility against Sotrovimab, a monoclonal antibody that targets a highly conserved epitope in the receptor binding domain (RBD) of SARS-CoV-2 [22]. Moreover, a recent multinational, placebo-controlled, randomized clinical ACTIV-3 trial investigated the neutralizing activity of sotrovimab and the two-antibody cocktails “BRII-196 plus BRII-198” in hospitalized COVID-19 patients, and showed no efficacy for improving clinical outcomes [29]. Therefore, the resistance phenomenon and discouraging clinical results led the authorities to remove its EUA (5 April 2022). Currently, the administration of BAM/ETE, CAS/IMD and sotrovimab is not recommended due to the lack of activity against the dominating omicron lineages. The only mAbs that preserve activity against predominant omicron variants are tixagevimab/cilgavimab and bebtelovimab as described in correspondence by Daichi Yamasoba and colleagues [30]. In fact, bebtelovimab is the only one that has shown remarkably preserved in vitro activity against all SARS-CoV-2 variants, including the omicron variant and the most recent dominant BA.4 and BA.5 subvariants [31]. The pan efficacy of bebtelovimab was also reported by the phase II BLAZE-4 trial (NCT04634409) that promoted the Emergency Use Authorization (EUA) for early treatment of COVID-19 outpatients by the FDA in February 2022. The EUA by EMA is ongoing. Finally, the use of bebtelovimab became important especially when antiviral nirmatrelvir-ritonavir is contraindicated [32,33]. Overall, mAbs reduced disease progression by approximately 70 to 85% against mild-to-moderate COVID-19 patients [19] but intravenous or subcutaneous administration represents a barrier to their use in a health care setting. However, constant monitoring of variants of concern is compulsory due to the rapid evolution of the epidemiological scenario.

### 3.2. Antiviral Agents

Since the beginning of the pandemic, antiviral drugs have been used in COVID-19 therapy for hindering the viral replicative cycle at different levels and in different ways. The available antiviral drugs target viral or host proteins necessary for virus burden persistence [9]. Overall, they are most effective in the early stages of the disease and are characterized by viral proliferation, unlike critical phenotype where inflammatory reaction dominates. Notably, oral antivirals should be delivered within five days after the onset of COVID-19 symptoms to ensure efficacy. Thus, their delayed administration easily results in the worst effectiveness [34].

#### 3.2.1. RNA-Dependent RNA Polymerase (RdRp) Inhibitors

The first licensed antiviral agents for COVID-19 were remdesivir and molnupiravir. They were approved for emergency use by the FDA to treat outpatients with mild COVID-19 phenotype and risk factors for progression to severe stages of the disease. Remdesivir is a non-canonical nucleotide able to stop the chain-elongation reaction of the viral RNA by RNA-dependent RNA polymerase (RdRp). Literature data gathered so far support the efficacy of intravenous remdesivir in reducing the risk and the length of hospitalization showing efficacy against several SARS-CoV-2 strains [35]. The “WHO Solidarity randomized trial” investigated the efficacy of remdesivir in inpatients with COVID-19. Notably, 8275 patients were randomized to remdesivir or its control; mortality and hospitalization were chosen as primary and secondary outcomes, respectively. Overall mortality was 602 (14.5%) in patients receiving remdesivir with respect to 643 (15.6%) in the control group. A sub-analysis focused on the disease severity revealed that among the severe COVID-19 patients, 151 (42.1%) of 359 in treatment with remdesivir died compared to 134 (38.6%) of 347 in the control group. Of the patients receiving oxygen support, 14.6% assigned to remdesivir died versus 16.3% assigned to control [36]. All these data showed a major efficacy of remdesivir in mild-to-moderate patients supporting the use of antivirals immediately following the onset of symptoms. Therefore, the current guidelines recommend remdesivir for unvaccinated ambulatory patients and vaccinated outpatients at risk for vaccine failure or at high risk for progression to severe disease within 7 days of symptom onset. The prodrug molnupiravir also inhibits RNA-polymerase of SARS-CoV-2 and other RNA viruses but presents a high mutational power in RNA strand, unlike remdesivir. Indeed, molnupiravir metabolization produces the cytidine nucleoside analogue N-hydroxycytidine (NHC) which is phosphorylated to the active form N-hydroxycytidine-5′-triphosphate (NHC-TP) into the cells. NHC monophosphate acts as a competitive substrate that will be incorporated by the SARS-CoV-2 viral RdRp introducing mutations (G to A and C to U substitution) in the viral genome and consequently inhibiting viral replication [37,38]. The literature reports a higher antiviral activity of molnupiravir than remdesivir, likely due to the high binding stability of NHC monophosphate to RNA viral which prevents its removal by exonucleases. EUA was based on data from 1734 randomized mild-to-moderate COVID-19 participants, recruited in phase III MOVe-OUT trial. The study investigated the efficacy and safety of molnupiravir (800-mg) compared to placebo, delivered within 5 days of the onset of symptoms. Molnupiravir showed superiority over placebo in preventing hospitalization or death (7.3% versus 14.1%, respectively) [39]. Nowadays, molnupiravir is licensed for adult unvaccinated outpatients and mild-to-moderate COVID-19 vaccinated patients at risk for vaccine failure, within 5 days from symptoms onset. However, molnupiravir use has not been recommended in international guidelines or is only recommended when no other treatment options exist due to its lower reported effectiveness at preventing hospitalization compared to other outpatient therapies.

#### 3.2.2. Protease Inhibitors

M protease (Mpro) and PL protease (PLpro), respectively known as nsp5 and nsp3, are essential enzymes in the virus replication cycle, representing a therapeutic target to prevent the success of the infection. They process the virus polyproteins into active proteins and differ from human proteases, promoting the lower toxicity of protease inhibitors [40]. Notably, nirmatrelvir (NM) is an oral protease inhibitor that is active by cleaving the 2 viral polyproteins; it is available in association with ritonavir^®^, a strong cytochrome P450-3A4 inhibitor, that increases nirmatrelvir concentrations reducing the dose regimen and the side effects of the antiviral. Recent reports found that nirmatrelvir-ritonavir (NM/r) reduced the risk of death and hospitalization, showing more efficacy than molnupiravir. Moreover, data derived from the EPIC-HR trial (Evaluation of Protease Inhibition for COVID-19 in High-Risk Patients) evaluating the efficacy of nirmatrelvir in non-hospitalized subjects without previous immunity against SARS-CoV-2 provided significant results on which the FDA EUA was based. The trial was performed when the delta variant was the predominant variant [41,42]. A real-world interesting study was next carried out, based on data obtained from electronic medical records of the Israeli population. The aim of this work was to assess the effectiveness of NM/r in reducing the rate of hospitalizations in COVID-19 subjects when the omicron variant was the most common variant. The results corroborated the efficacy of NM/r in preventing severe COVID-19 onset, above all among adults 65 years of age or older [43]. Overall, all these studies brought to light the importance to start NM/r treatment in early COVID-19 to prevent the worsening of the illness towards severe disease and quickly reduce SARS-CoV-2 viral load. Unfortunately, viral mutations represent the hot-spot for achieving successful therapy, probably responsible for the virological and clinical rebound in patients receiving NM/r as described by Charness et al. [44]. Notably, the mechanisms involved in COVID-19 recrudescence after NM/r treatment were investigated, suggesting that reduced target drug concentrations related to pharmacokinetic changes and/or insufficient therapy length were involved [45]. Moreover, some mutations in the Mpro have been revealed such as alanine 260 to threonine (A260T) or valine (A260V) in samples of patients receiving NM/r but did not cause a reduction in Mpro activity [46]. Overall, the occurrence frequency of rebound illness is unclear, but increases in viral load were detected in 1 to 2% of participants in the phase III clinical trial [47]. However, the mechanisms of COVID-19 recrudescence after treatment are still to be investigated. Currently, NMV/r is licensed for adult unvaccinated outpatients and vaccinated outpatients at risk for vaccine failure and/or progression to severe disease within 5 days of symptom onset.

#### 3.2.3. Drugs for COVID-19 Immune System Regulation

In response to viral replication, the immune system spurs effectors such as tumor necrosis factor (TNF)-α, interferon (IFN) and several interleukins (IL-1β, IL-6). In turn, a chain reaction is triggered in an attempt to resolve the infection. In particular, type-I IFN activates the janus kinase/signal transducer and activator of transcription (JAK/STAT) pathway, crucial for protecting against viral progression. The JAK/STAT axis is involved in cell proliferation and differentiation, apoptosis and the physiological process of the immune system [48,49,50]. JAK/STAT is also implicated in the aberrant immune response in SARS-CoV-2-infected respiratory epithelial cells with the activation of CD4+ and CD8+ positive T cells, NK cells, monocytes and the release of high levels of pro-inflammatory cytokines, representing an attractive target in COVID-19 patients. Furthermore, JAK-STAT activation promotes the senescence of SARS-CoV-2 infected cells, perpetuating inflammation. JAK inhibitors such as Baricitinib, Ruxolitinib and Tofacitinib have been developed. Baricitinib is an oral selective JAK1/JAK2 inhibitor that may block viral entry into host cells, prevent a cytokine storm, and resolve immune dysregulation in patients with SARS-CoV-2 pneumonia. An additional mechanism related to baricitinib is AP2-associated protein kinase 1 (AAK1)/cyclin G-associated Kinase (GAK) inhibition that contributes to preventing SARS-CoV-2 entry and intracellular virion assembly [51]. Several studies assessed the efficacy of baricitinib as the observational, longitudinal trial (IMMU-NOVID) (NCT04438629) which observed a significant reduction in IL1β, IL6, TNFα, recovery of circulating T and B cells and increased antibody production against the spike protein following baricitinib delivery. This was associated with a reduction in the need for oxygen therapy, pointing out the capacity of baricitinib of preventing disease progression [52]. Baricitinib reduced SARS-CoV-2 viral load, COVID-19 mortality rate, and intensive care unit admissions of COVID-19 pneumonia in a retrospective study (NCT04358614) [53]. A randomized phase III trial (COV-BARRIER, NCT04421027) described a significantly reduced mortality at 28 days and 60 days after baricitinib plus SOC. Finally, the clinical trial (NCT04401579, ACTT-2) found that the association baricitinib with remdesivir was superior to remdesivir alone in reducing the median time to recovery, and accelerating improvement in clinical status at day 15 but did not detect greater differences in mortality [54]. An ACTT-4 trial evaluated the association of baricitinib-remdesivir compared to dexamethasone-remdesivir, finding a significant efficacy for both treatments. Based on these results, baricitinib was approved by FDA for EUA along with remdesivir; moreover, it is strongly recommended for severe cases, especially in patients receiving corticosteroids baseline [55]. Baricitinib is also licensed as an alternative treatment to the IL-6 inhibitor tocilizumab if the latter is not available. Tocilizumab is able to block the IL-6 soluble and membrane receptor, approved for severe forms of COVID-19, characterized by the overproduction of proinflammatory cytokines. Recently, large-scale clinical trials and a meta-analysis demonstrated that Tocilizumab was beneficial in critical COVID-19 patients. In particular, the RECOVERY and REMAP-PAC trials analyzed the impact of tocilizumab in patients with clinical evidence of progressive COVID-19, discovering the ability of tocilizumab to improve survival and decrease the need for mechanical ventilation and mortality. The beneficial effects were more significant when tocilizumab was combined with corticosteroids [56,57].

## 4. Therapeutic Agents in Clinical Trials

Nowadays, the attempts to curb the virus spread and the onset of serious cases are still ongoing, thus the potential therapeutic effects of many drugs is being investigated by several clinical trials.

### 4.1. Therapeutic Agents in Phase III Clinical Trials

#### 4.1.1. RNA-Dependent RNA Polymerase (RdRp) Inhibitors

**Figure 2:**
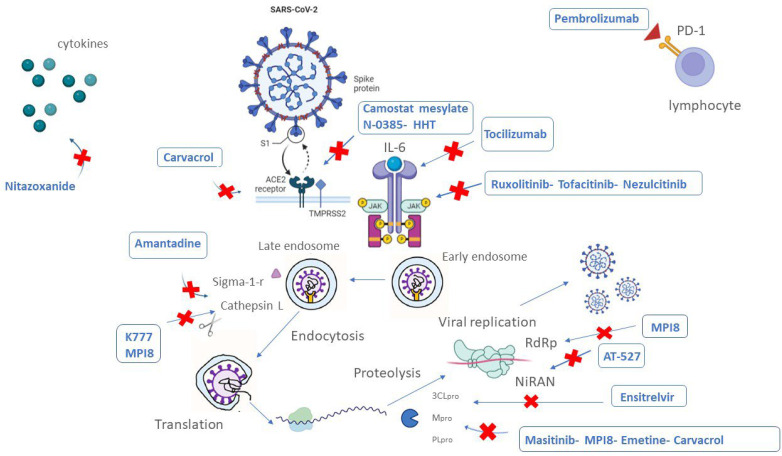
An illustrative picture showing the drugs/molecules in clinical and preclinical phase for COVID-19 therapy and their related targets.

Interesting results from the use of remdesivir and NM/r have led researchers to identify new molecules able to modulate RdRp. Unfortunately, clinical data have not always provided results promoting their use. For instance, favipiravir is a nucleoside analogue in the form of adenine or guanine derivatives that selectively binds to RdRp and causes lethal mutagenesis upon incorporation into the virus RNA. Similar to remdesivir and molnupiravir, it inhibits viral RNA synthesis. The phase III clinical trial PRESECO (PREventing SEvere COVID-19) studied favipiravir in non-hospitalized COVID-19 patients, reporting no statistical significance on the time to sustained clinical recovery (primary outcome) (Table 1). Analyses of the key secondary endpoints also failed to reach statistical significance [58,59]. In fighting the pandemic, not only repurposing drug approaches but also the identification of new compounds is crucial. In this context, the guanosine analog AT-527 represents a potential candidate against SARS-CoV-2. Recent studies identified AT-527 and its triphosphate metabolite, AT-9010, as a dual inhibitor of both RdRp and nidovirus RdRp-associated nucleotidyl transferase (NiRAN) (Figure 2). Notably, AT-9010 is incorporated into viral RNA, induces chain-elongation ending and inhibits nucleotidyl transferase activity of NiRAN, showing a pleiotropic action against mutation onset. AT-527 recently entered phase III clinical trials to evaluate the time to alleviation or improvement of COVID-19 symptoms compared to placebo in the outpatient setting (NCT04889040) (Table 1). The recruitment is terminated and the results are under review [60].

#### 4.1.2. Protease Inhibitors

Beyond RdRp, proteases remain one of the main targets to be modulated, thus new compound targeting them have been identified. s217622 (ensitrelvir) is a novel selective inhibitor of the 3C-like protease of SARS-Cov-2 (Figure 2), essential in viral replication. Safety, tolerability and pharmacokinetics of different oral doses of ensitrelvir were assessed. It was well tolerated with a long half-life that supported once-daily oral dosing [61]. Based on these data, further studies on ensitrelvir were performed and a clinical phase III is ongoing. The main aim of this study is to evaluate the efficacy of oral ensitrelvir versus placebo in non-hospitalized COVID-19 subjects not receiving SOC. The primary outcome is the median time to sustained symptom resolution and the estimated study completion date is August 2023 (NCT05305547) (Table 1).

#### 4.1.3. Drugs for COVID-19 Immune System Regulation

The license of baricitinib in anti-COVID therapy promoted the research for other potential JAK inhibitors such as Ruxolitinib. It is a JAK1/JAK2 inhibitor (Figure 2) that exerted potential benefits for severe COVID-19 disease. Due to the positive results of multicenter randomized trial that reported a faster clinical improvement in patients receiving Ruxolitinib with the SOC compared with the SOC group alone [62], a phase III clinical trial was performed. The latter is a randomized, placebo-controlled study which analyzed the efficacy of Ruxolitinib in COVID-19-associated acute respiratory distress syndrome (ARDS) who require mechanical ventilation, showing a reduction in overall mortality, number of ventilators and oxygen-free days (Table 1). To obtain the best result, it is useful to start treatment immediately after respiratory symptoms. Moreover, tofacitinib, a selective inhibitor of JAK2 (Figure 2), was investigated in a randomized, placebo-controlled study (STOP-COVID, NCT04469114) (Table 1), finding a significant reduction in death or respiratory failure in patients hospitalized with COVID-19 pneumonia compared with the placebo [63].

Since the onset of severe cases is the most difficult obstacle to overcome, several clinical studies focus on the therapeutic management of hospitalized patients. As mentioned earlier, one of the immunophenotype related to severe forms of COVID-19 is the overproduction of proinflammatory cytokines and a beneficial approach to cope with COVID-19 illness is blocking the hypercytokinemia. The cytokine storm and the related systemic organ damage can be modulated by nitazoxanide (NTZ) (Figure 2) due to its ability to inhibit the production of pro-inflammatory cytokines and induce interferon-stimulated viral gene expression. Many clinical trials are investigating the efficacy and safety of NTZ; notably a phase III clinical trial tested NTZ activity and safety in early COVID-19 patients, showing its capacity to hinder clinical deterioration and reduce the time to sustained clinical response and the rate of progression compared to placebo (NCT04486313) (Table 1) [64]. Interestingly, NTZ seems to be associated with a low rate of viral resistance. In conclusion, NTZ could play an important role in reducing the number of severe illnesses and hospitalizations.

#### 4.1.4. Other Therapeutic Agents

Antiviral activity towards SARS-CoV-2 was also investigated by CO-PREVENT study (NCT04854759) (Table 1) that analyzed the efficacy of oral amantadine in mild COVID-19 patients in preventing progression toward severe stages of the disease. The endpoints of the study are disease worsening, time to clinical deterioration, overall survival and quality of life, but the results are not yet posted [65]. Several other phase III clinical trials focused on the efficacy of amantadine in the early stages of COVID-19 are in recruitment status (NCT04894617, NCT04952519, NCT05504057) (Table 1). All these trials are supported by several hypothesized mechanisms of action of amantadine such as antagonism of cathepsin L, the Sigma-1 receptor and a modulation of neurotransmitters (Figure 2).

### 4.2. Therapeutical Agents in Phase II Clinical Trials

#### 4.2.1. Protease Inhibitors

A recent phase II clinical trial (NCT05047783) (Table 1) has been testing the efficacy of M^pro^ inhibitor Masitinib, at three different dosages (last update of 23 June 2022). The ability of Masitinib in inhibiting M^pro^ (Figure 2) and reducing viral shedding of SARS-CoV-2 was investigated once again in patients with symptomatic mild-to-moderate COVID-19, supporting the use of oral antiviral agents in the early disease [66]. Many other clinical trials are focused on molecules targeting host genes since these latter have a low propensity to mutate than viral genes. Hence, antiviral drugs targeting host proteins may offer a good option compared to those targeting viral proteins. In fact, the transmembrane protease serine 2 (TMPRSS2) represents a potential therapeutic target [67]. TMPRSS2 mediated one of the two proteolytic cleavages involved in membrane fusion following ACE-2/spike interaction. TMPRSS2 inhibition could represent a strategy to block viral entry. Camostat mesylate (CM), an inhibitor of TMPRSS2 (Figure 2), was tested in the randomized “Austrian Coronavirus Adaptive Clinical Trial” (ACOVACT), showing its capacity to significantly reduce the progression to mechanical ventilation or death and the time to clinical improvement in hospitalized patients compared to lopinavir/ritonavir (NCT04351724) (Table 1) [68]. Moreover, calpain is another host protein whose inhibition could lead to the suppression of viral entrance via endocytosis, maturation of the primary endosome to the secondary endosome and virus replication. In a mouse model of bleomycin lung injury (BLD-2660), an oral selective inhibitor of calpain (CAPN) 1, 2 and 9 called BLD-2660 (Figure 2), reduced IL-6 levels in bronchoalveolar lavage fluid and attenuated fibrosis damage [69]. Based on these data, a randomized phase II, placebo-controlled study assessed the efficacy of BLD-2660 in hospitalized COVID-19 subjects, assuming its ability to downregulate IL-6 and reduce potential long-term fibrosis and loss of pulmonary function resulting from SARS-CoV pneumonia (NCT04334460) (Table 1).

#### 4.2.2. Drugs for COVID-19 Immune System Regulation

The efficacy of pan-JAK inhibitor Nezulcitinib (Figure 2) was investigated in hospitalized patients with confirmed COVID-19-associated acute lung injury and impaired oxygenation (NCT04402866) (Table 1). Overall mortality associated with Nezulcitinib was only 5% at day 28 compared to 33% in the placebo group [70]. The aberrant immune system always develops in severe cases of COVID-19 by different phenotypes. Several severe cases of COVID-19 present a neutrophil increase and a decrease in CD4+ T cells leading to an immunosuppressive microenvironment [71]. Lymphopenia is associated with T cell exhaustion, both due to apoptosis mediated by a virus-induced overexpression of type 1 programmed death receptors (PD-1) and its ligand (PD-L1) [72,73]. Interestingly, cancer patients receiving checkpoint inhibitors are less prone to develop COVID-19 disease compared with those receiving chemotherapy, pointing out the immunosuppressive nature of COVID-19 infection [74]. Hence, the T cells reactivation by checkpoint inhibitors could represent a valid approach to counter COVID-19 infection [75]. Moreover, a PD-1 upregulation in CD4+ and CD8+ T cells was observed in COVID-19 disease, supporting the use of checkpoint inhibitors in reinforcing viral clearance [76]. The COPERNICO trial investigated the ability of Pembrolizumab (anti-PD-1 antibody) plus tocilizumab in interrupting hyperinflammation and restoring cellular immunocompetence (Table 1). The addition of pembrolizumab/tocilizumab to SOC reduced the hospitalization period, with higher and faster discharges than SOC alone (NCT04335305).

## 5. Preclinical Studies

While clinical trials in advanced stages are ongoing, preclinical studies have been started with the aim to test new molecules and new pathways to be modulated. Since SARS-CoV-2 may also enter the human cells through the clathrin-mediated endocytosis, overwhelming TMPRSS2 pharmacological block, the research identified a more potent inhibitor of TMPRSS2 (N-0385) (Figure 2). Interestingly, intranasal administration of N-0385 exerted complete protection against SARS-CoV-2-induced mortality in the K18-hACE2 mouse model and used as an ideal model of severe COVID-19, representing a valid prophylactic and therapeutic option against emerging SARS-CoV-2 variants [77]. However, when TMPRSS2 concentration near the ACE-2 receptor/SARS-CoV-2 complex is insufficient, inhibition of the cathepsin and calpain-mediated entry pathway might provide an alternative therapeutic strategy. In fact, activation of SARS-CoV-2 spike protein by Cathepsin L is one of the crucial steps that assists the coronavirus uptake and its inhibition blocks viral RNA genome entry into the host cytoplasm [78]. Zhu J. et al. investigated a potential anti-SARS-CoV-2 activity of a novel class of self-masked aldehyde inhibitors (SMAIs). They characterized the human cathepsin L inhibition of these compounds and found that propargyl analogue of K777 significantly blocked the SARS-CoV-2-induced cytopathic effect in Vero E6 and A549/ACE2 cells [79]. A therapeutic approach leading to a decreased infection rate and improved efficacy could be a synergistic block of crucial factors in the viral replication cycle. A molecular screening performed by Xinyu R. Ma et al. identified MPI8 as a potential dual candidate for M^pro^ and cathepsin L inhibition (Figure 2), paving the way for preclinical and clinical investigations for treating COVID-19 [80]. Cathepsin L and B can also be modulated by amantadine (Figure 2) [81,82]. The unmistakable importance of the spike−ACE2 interaction in blocking viral replication prompted Tedesco et al. to develop small molecules able to target protein−protein interactions. A pool of synthesized peptidomimetics were identified with a great inhibition power of the spike–ACE2 interaction, paving the way for the development of new therapeutics against coronavirus infections [83].

## 6. Natural Products and Metal-Based Drugs as Adjuvants Agents in COVID-19 Control

To curb newly emerging SARS CoV2 variants, many plant metabolites may be valid alternatives against SARS-CoV-2. Among natural metabolites, alkaloids have potential drug activity by intercalation power against nucleic acids (DNA or RNA), stabilizing them in single-stranded form. Homoharringtonine (HHT) is a cytotoxic plant alkaloid [84] strongly targeting the mRNA translation; its interesting antiviral activity promoted a protocol for clinical trials of HHT nebulization on COVID-19 patients has been registered (ChiCTR-2100045993) by the Ditan Hospital [85]. Moreover, clinical trials and observational studies showed the positive effects of alkaloids colchicine and emetine in COVID-19 subjects [86,87,88,89]. Carvacrol (CARV) is an essential oil extract derived from different plants which exerted antioxidant, antiviral and Ca^2+^ influx modulator activities. Notably, Javed et al. reported a potential action in COVID-19, showing its potential inhibition activity of ACE2 and M^pro^ with a significant block in the host cell entry and replication of SARS-CoV-2 (Figure 2) [90,91]. Different research groups discovered the in vitro and in vivo anti-SARS-CoV-2 activity of cepharanthine, a natural alkaloid extracted from Stephania japonica. Cepharanthine have antioxidant and anti-inflammatory properties [92,93] and could exert an antiviral effect by modulating Hypoxia-inducible factor-1 (HIF-1), a dysregulated factor in COVID-19 [94]. Recently, a high antiviral activity of cepharanthine against SARS-CoV-2 was identified, also preserving antiviral action against the Beta (B.1.351) variant. Thus, a phase II clinical study (NCT05398705) of cepharantine was completed in mild COVID-19 patients but the results are not posted yet. Finally, the LINCOLN survey showed the beneficial effects of Vitamin C and L-Arginine in long-COVID [95,96,97] with significantly less severe long-COVID symptoms and a better effort perception when compared to the alternative treatment group [98]. Overall, natural products represent an important tool in fighting COVID-19 but their use is only recommended in mild-to-moderate stages of the disease. Similarly, natural products as well as metal-based molecules may be valid tools for combating COVID-19. Phase II clinical trials are ongoing to evaluate the efficacy of ebselen (SPI-1005) in mild and severe COVID-19 patients (NCT04484025/NCT04483973). Ebselen is a synthetic organoselenium with a glutathione peroxidase function which exhibits in vitro antiviral activity against SARS-CoV-2 [99,100,101]. Iron level alterations are also detected in COVID-19 patients, related to oxidative stress. Therefore, iron chelators such as deferasirox, deferoxamine and deferiprone could be beneficial in decreasing cytotoxicity and oxidative stress damage associated with hyperferritinemia [102]. Among them, deferiprone seems to be the most potent antioxidant drug in vitro, in vivo and in clinical models of COVID-like diseases, supporting its potential therapeutical use [103,104].

## 7. Delivery Strategies to Improve COVID-19 Therapy Management

Since the primary target of SARS-CoV-2 replication is the lung, the inhalation delivery of drugs could represent the best route of administration to cope with COVID-19. In fact, several lung diseases, such as asthma and chronic obstructive pulmonary disease (COPD) were treated by inhaler drugs that reach a maximum pharmacological effect with minimum systemic exposure [105,106]. Moreover, many drugs are poorly soluble in water and/or susceptible to denaturation of the protein active ingredient [107,108,109,110,111,112], thus different formulations have been prepared to enhance their solubility and reduce denaturation. Hence, testing new formulations for these drugs has become essential in order to boost the local antiviral activity and provide a suitable alternative. Currently, there are several inhaled therapeutics being tested for COVID-19. Remdesivir was formulated as a dry powder formulation using nanoliposomes for inhalation by Vartak et al., resulting in prolonged drug release and reduced frequent dosing [113,114]. Several other clinical trials are testing the inhaled formulations of drugs: the INHALE-HEP trial (NCT0463524) and NOVATION-1 (NCT04669015). The first investigated the efficacy of inhaled nebulized unfractionated heparin (UFH) in hospitalized patients [115,116,117]; the second investigated the safety and efficacy of Novaferon (recombinant IFN-α-like protein) in hospitalized patients [118,119]. Overall, the large number of clinical trials designed to test the efficacy of inhaled strategies of various drugs offers a promising approach to better managing the outbreak evolution.

## 8. Conclusions

The main goal of anti-COVID therapy is to lower the circulating viral load and hinder severe symptom onset to reduce the risk of hospital admission and death, and consequently reduce pressure on healthcare systems. Since most of the drugs tested experienced significant changes due to the rapid evolution of the disease and most trials addressed severe COVID-19 patients, it is necessary to organize more clinical trials addressing drugs for prophylaxis and the early stages of the disease to prevent progression toward more severe stages. Therefore, the availability of safe oral drugs is the first step to overcoming the COVID-19 outbreak. However, it is essential to continuously promote an update of pharmacological treatment due to the evolution of COVID-19 epidemiology. In conclusion, the implementation of therapeutic approaches with better management of COVID-19 is needed to synergize vaccine efficacy. Thus far, one solution to reduce drug resistance is “cocktail” therapy with the potential to lead us to win this battle.

## Figures and Tables

**Table 1:** Summary of clinical trials of therapeutics for SARS-CoV-2, registered on ClinicalTrials.gov.

Drug	Target	NCT	Clinical Status	Study Design	Treatment	Primary Outcome
Favipiravir	RdRp	NCT04600895	Phase III Completed	Double-blinded, randomized study	Favipiravir vs. Placebo	Time (0–28 days) to sustained clinical recovery. The endpoint will be positive when the subject has reached sustained alleviation of symptoms.
AT-527	RdRp/NiRAN	NCT04889040	Phase III Terminated	Multicenter, randomized, double-blind study	AT-527 vs. Placebo	Time (0–29 days) to alleviation or improvement of COVID-19 symptoms, evaluated by COVID-19 Symptom Diary.
S-217622 (Ensitrelvir)	3C-like protease	NCT05305547	Phase III Recruiting	Multicenter, randomized, double-blind, 24-week study	S-217622 vs. placebo	Median time (0–29 days) to sustained symptom resolution.
Amantadine	Cathepsin L Agonism of Sigma-1 receptors Modulation of neurotransmitters	NCT04854759	Phase III Recruiting	Multicenter randomized, double-blind, non-commercial study	Amantadine Hydrochloride vs. Placebo	Development of clinical deterioration (0–15 days), defined as dyspnoea, physical examination, doctor’s assessment; Clinical deterioration occurs, defined as drop in O2 saturation and achievement of ≥4 points on the WHO scale.
Amantadine	Cathepsin L Agonism of Sigma-1 receptorsModulation of neurotransmitters	NCT04894617	Phase III Recruiting	Randomized, double-blinded, placebo-controlled, single center study	Amantadine vs. Lactose monohydrate	Clinical status on day 14 according to 8 point ordinal scale for clinical improvement.
Amantadine	Cathepsin L Agonism of Sigma-1 receptors Modulation of neurotransmitters	NCT04952519	Phase III Recruiting	Randomized, parallel assignment, triple-blinded study	Amantadine vs. Placebo	Time to recovery (defined as the first day during the 28 day clinical follow-up during which the patient’s clinical condition is graded 1, 2 or 3 on an eight-point “Normal Symptom Score”)
Amantadine	Cathepsin L Agonism of Sigma-1 receptorsModulation of neurotransmitters	NCT05504057	Phase IV Recruiting	Observational, case–control, Retrospective study	Amantadine vs. Antihistamine	Rate of hospital admissions (time frame: from March 2020)
Ruxolitinib	JAK1/JAK2	NCT04377620	Phase III Terminated	Randomized, double-blind, multicenter study	Ruxolitinib vs Placebo	Overall mortality (percentage of participants who have died due to any cause during a time frame from 0 day to day 29).
Tofacitinib	JAK2	NCT04469114	Phase III Completed	Multicenter, randomized, double-blinded, parallel-design study	Tofacitinib: vs. Placebo	Death or respiratory failure * until day 28
Nitazoxanide	- inhibition of inflammatory cytokines - induction of interferon-stimulated viral gene expression.	NCT04486313	Phase III Completed	Multicenter, randomized, double-blinded study	Nitazoxanide vs. Placebo	Reducing the Time to Sustained Response (0–21 days)
Masitinib	M^pro^	NCT05047783	Phase II Recruiting	Randomized, double-blinded, placebo-controlled study	Masitinib Mesylate Vs. Palcebo	SARS-Cov-2 viral load at day 10 (time-weighted average change from baseline in viral shedding)
**Camostat mesylate**	TMPRSS2	NCT04351724	Phase II/III Recruiting	Randomized, controlled, multicenter, open-label basket study	Camostat mesylate vs. Lopinavir/Ritonavir	Clinical improvement defined as time from randomization to an improvement of at least one category measured on a seven-category ordinal scale (proposed by WHO).
**BLD-2660**	calpain (CAPN) 1, 2, and 9	NCT04334460	Phase II	Randomized, double-blinded study	BLD-2660 vs. Placebo	Time to Recovery (0–28 days) defined by no longer requiring oxygen support or hospital discharge, whichever occurs first.
**Nezulcitinib**	pan-JAK inhibitor	NCT04402866	Phase II Completed	Randomized, double-blinded, parallel-group, multicenter study	Nezulcitinib vs. Placebo	Number of Respiratory Failure-free Days (RFDs) from randomization to day 28. An RFD was defined as a day that a participant was alive and did not require the use of any respiratory support.
**Pembrolizumab/Tocilizumab**	PD-1/IL-6	NCT04335305	Phase II Terminated	Randomized, controlled, open-label study	Pembrolizumab/Tocilizumab vs. SOC	Percentage of patients with normalization of SpO2 ≥ 96% on room air (time frame from treatment initiation to day 14) assessed by hospital records.

* Respiratory failure requiring hospitalization for: invasive mechanical ventilation; non-invasive ventilation or high-flow oxygen device; requiring supplemental oxygen; requiring ongoing medical care.
